# Incidence and risk factors of nasogastric feeding intolerance in moderately-severe to severe acute pancreatitis

**DOI:** 10.1186/s12876-022-02403-w

**Published:** 2022-07-02

**Authors:** Jiajia Lin, Cheng Lv, Cuili Wu, He Zhang, Zirui Liu, Lu Ke, Gang Li, Zhihui Tong, Jianfeng Tu, Weiqin Li

**Affiliations:** 1 Center of Severe Acute Pancreatitis (CSAP), Department of Critical Care Medicine, Jinling Hospital, Medical School of Nanjing University, No. 305 Zhongshan East Road, Jiangsu, Nanjing, 210002 People’s Republic of China; 2 Department of General Surgery, Jinling Hospital, Medical School of Nanjing University, Nanjing, 210002 People’s Republic of China; 3 Department of Critical Care Medicine, Jinling Hospital, Medical School of Southeast University, Nanjing, 210002 People’s Republic of China; 4 Emergency and Critical Care Center, Department of Emergency Medicine, Zhejiang Provincial People’s Hospital, People’s Hospital of Hangzhou Medical College, Hangzhou, 310014 Zhejiang People’s Republic of China; 5 National Institute of Healthcare Data Science, Nanjing University, Nanjing, China

**Keywords:** Enteral nutrition, Feeding intolerance, Acute pancreatitis, Jejunal feeding, Prognosis

## Abstract

**Background:**

The importance of enteral nutrition (EN) in acute pancreatitis (AP) has been emphasised. Nasogastric (NG) feeding has been the preferred route for EN delivery in most AP patients intolerant to oral intake. However, gastric feeding intolerance (GFI) was frequently reported, especially in patients with more severe diseases. This study aimed to investigate the incidence and risk factors for GFI in moderately-severe to severe AP.

**Methods:**

This is a single-centre, retrospective study. All the data were extracted from an electronic database from April 2020 to May 2021. Data were prospectively collected during hospitalisation. Patients diagnosed with moderately-severe to severe AP and admitted within seven days from the onset of abdominal pain were assessed for eligibility. Patients who showed signs of intolerance to gastric feeding and required switching to nasojejunal (NJ) feeding were deemed GFI. Multivariable logistic regression was performed to assess potential risk factors of GFI.

**Results:**

A total of 93 patients were analysed, of whom 24 were deemed GFI (25.8%), and the rest tolerated NG feeding well (*n* = 69). In patients with GFI, the median time of switching to NJ feeding was five days (interquartile range: 4–7 days) after admission. The multivariable analysis showed that respiratory failure (odds ratio = 3.135, 95% CI: 1.111–8.848, *P* = 0.031) was an independent risk factor for GFI.The mean daily energy delivery in the following three days after switching to NJ feeding was significantly higher than the first three days after initiation of NG feeding in patients with GFI [920.83 (493.33–1326) vs. 465 (252.25–556.67) kcal, *P* < 0.001].

**Conclusion:**

GFI is common in moderately-severe to severe AP patients with an incidence of 25.8%, and the presence of respiratory failure may increase the risk of GFI.

**Supplementary Information:**

The online version contains supplementary material available at 10.1186/s12876-022-02403-w.

## Introduction

The clinical superiority of enteral nutrition (EN) over total parenteral nutrition (TPN) has been well proved in acute pancreatitis (AP). For the route of EN delivery, several randomised controlled trials had shown that there was no difference in terms of mortality, infectious complications, length of hospital stay, or energy balance between nasogastric (NG) and nasojejunal (NJ) feeding in patients with severe acute pancreatitis (SAP) requiring tube feeding [1–4]. Given the technical difficulty for NJ tube insertion, NG feeding has been recommended as the primary choice for SAP patients [5].

Recently, ESPEN guidelines recommend initial EN delivery via a NG tube in AP patients requiring tube feeding, and the NJ route should be preferred in case of intolerance [6]. In patients with gastric feeding intolerance (GFI), switching to NJ feeding is the alternative option [7]. However, due to the time required for evaluating GFI and NJ tube placement, this switch would inevitably weaken the benefits of early EN [8–10], leading to an energy deficit [11]. Moreover, the risk of aspiration accompanying NG feeding, a common clinical finding in patients with more severe AP, can not be ignored, especially in patients with increased abdominal pressure [12, 13]. Therefore, NJ feeding as the primary choice may provide clinical benefits in patients with a high risk of GFI and aspiration.

In this study, we aimed to investigate the incidence of GFI and assess potential risk factors for GFI in moderately-severe to severe AP patients. The results of the current study could provide preliminary data for future studies.

## Methods

### Study population

This study is a single-centre, retrospective study conducted in the Centre of Severe Acute Pancreatitis, Jinling Hospital. All the data were extracted from the Acute Pancreatitis Database, which was approved by the institutional review board of Jinling Hospital (2019NZKY-003–01). Informed consent involving data storage and academic use of data was obtained from each patient during hospitalisation. Clinical data were collected prospectively during hospitalisations. All consecutive patients with a primary diagnosis of AP admitted from April 2020 to May 2021 were screened for eligibility. Patients were included if they were diagnosed with moderately-severe to severe acute pancreatitis, and admitted within seven days from the onset of abdominal pain. Patients who stayed in the hospital less than three days, were impossible for EN or received NJ feeding before admission were excluded.

### Nutrition practise

EN was initiated within 24–48 hours after admission if oral intake is not possible. Nasogastric feeding was the primary route for EN, while NJ feeding was only implemented in patients who could not tolerate gastric feeding. Intolerance of gastric feeding and switching to NJ feeding were considered if the patients met at least one of the following criteria: 1) enteral feeding has to be suspended for the gastrointestinal symptoms, such as large gastric residual volumes (GRV>500 ml/6h), abdominal pain, or vomiting due to delayed gastric emptying, 2) failed to reach the target (70% of the estimated target) within 72 hours of EN initiation [14]. The final decision regarding the requirement of NJ feeding depends on the treating physician. The method for NJ tube placement also at the discretion of the treating physician (endoscopic technique or bedside ultrasound-assisted technique), which was described detailedly in our previous study [15]. After that, an abdominal radiograph was used to confirm if the tip of the NJ tube had passed the ligament of Treitz into the mid-distal jejunum.

### Definitions and outcomes

Time to discharge alive from the hospital within 30 days after hospital admission and other clinical outcomes were compared between patients with and without GFI [16, 17]. The diagnosis of AP required two of the following three features: (1) abdominal pain consistent with acute pancreatitis, (2) serum lipase activity or amylase activity at least three times greater than the upper limit of normal, and (3) characteristic findings of acute pancreatitis on computed tomography. The severity of AP was categorised as mild (MAP), moderately-severe (MSAP), and severe (SAP) according to the Revised Atlanta criteria (RAC) [18]. The energy target is reaching at least 70% of the energy requirements. The energy requirements were estimated based on the weight-based Eq. (25 kcal/kg of body weight on hospital admission). Respiratory failure in this study was defined as a modified Marshall score of ≥ 2 (PO2:FiO2 ratio less than 300) according to the RAC.

### Data collection

Data concerning demographic and baseline clinical characteristics, including age, gender, etiologies, body mass index (BMI), complications, and clinical scores like Acute Physiology and Chronic Health Evaluation II (APACHE II) score [19] at admission were extracted from the database. Nutrition therapy variables, including time to switching to NJ feeding (in days), the daily amount of energy delivery after feeding tube placement, and intolerance symptoms, were collected from medical records. The local and systemic complications, including pancreatic necrosis, respiratory failure, acute kidney injury (AKI), shock during the hospital stay, were also collected.

### Statistical analysis

Data were analysed using SPSS for Windows version 18 (SPSS Inc., Chicago, IL, USA). The distribution of continuous variables was examined for normality using the Shapiro–Wilk test. Continuous variables were expressed as median (interquartile range) and analysed by Mann–Whitney U test. The difference in the amount of energy delivery between pre-switch and post-switch in the GFI group was compared using the paired Wilcoxon test. Categorical variables were expressed as absolute numbers (percentage) and compared by Pearson's chi-square or Fisher exact test as indicated. APACHE II score was dichotomised into two groups (< 8 and ≥ 8). Covariates including age, gender, respiratory failure, etiology, and APACHE II were tested by univariate analysis to examine the association between clinical variables and GFI. Parameters with *P* < 0.1 in the univariate analysis would enter the multiple logistic regression analysis to identify the risk factors for GFI. Pearson's correlation coefficient and the variance inflation factor were used to detect the presence of multicollinearity among variables included in the regression model. The VIF > 5 was considered as an indicator of multicollinearity [20, 21]. Kaplan–Meier methods were used to display curves for time to discharge alive from the hospital within 30 days after hospital admission. A log-rank test was conducted to compare the survival curves of the GFI group and non-GFI group. Sensitivity analyse was used to evaluate the robustness of our findings via propensity score matching (PSM). One-to-one nearest neighbour matching with a caliper width of 0.3 was used in the PSM and the standardised mean difference (SMD) were calculated for variables (age, gender, etiology and time from the onset of abdominal pain to hospital admission) before and after propensity score matching. A difference with a two-tailed *P* < 0.05 is considered statistically significant.

## Results

### Patients enrolment and characteristics

**Fig. 1:**
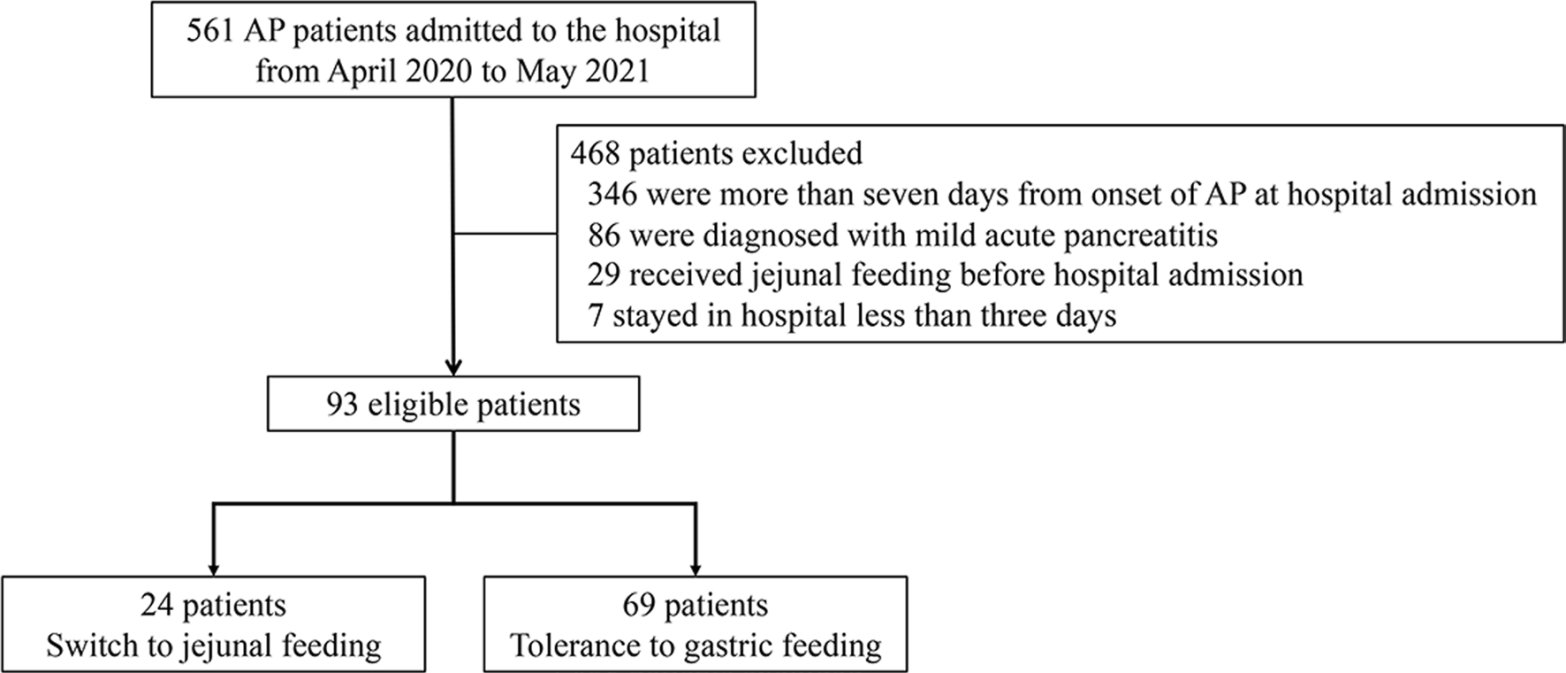
Flowchart of patient selection

A total of 561 patients were screened, and 468 patients were excluded (Fig. 1). Of the 93 eligible patients, 24 patients (25.8%) were switched to NJ feeding due to intolerance of gastric feeding (GFI group). Table 1 describes the baseline and demographic characteristics of the study cohort. The median time from the onset of abdominal pain to study hospital admission was three days in the study cohort. The majority of the study subjects (88/93, 94.6%) were referrals and the time from the onset of abdominal pain to initial hospital admission was within 24 h in most cases except two (on the second day). Hypertriglyceridemia and biliary origin were the two most common causes. The majority of the study subjects had pancreatic necrosis at hospital admission. A comparison of clinical characteristics and outcomes between the GFI and the non-GFI patients is shown in Table 2. There were no significant differences in age, gender, BMI, and etiologies between groups. Patients in the GFI group had a higher APACHE II score at admission [10(7–11) vs. 7(5–10), *P* = 0.003] compared with the non-GFI group. A higher proportion of patients with systemic complications (respiratory failure and AKI) at hospital admission was observed in GFI patients. For clinical outcomes, the GFI patients had a lower energy target-reaching rate at day3-7 after EN initiation, higher incidence of infected pancreatic necrosis, and more extended hospital stay. Only one patient died within 30 days after ICU admission in the GFI group. Kaplan–Meier survival curves illustrated that time-to-discharge alive from the hospital was shorter in patients without GFI (Additional file 1: Fig. S1).

**Table 1:** Baseline and demographic characteristics of the study cohort

Variables	Total *N* = 93
Age	40 (33–48)
Male	63 (67.7)
BMI	26.1 (24.1–29.4)
Time from onset of abdominal pain to study hospital admission	3 (2–5)
RAC	
Moderate	68 (73.1)
Severe	25 (26.9)
Etiology	
Hypertriglyceridemia	43 (46.2)
Biliary	36 (38.7)
Others	14 (15.1)
APACHE II	7 (5–10)
Presence of pancreatic necrosis	68 (73.1)
Systemic complications at hospital admission	
Respiratory failure	39 (41.9)
AKI	7 (7.5)
Shock	3 (3.2)
Duration of respiratory failure < 48 h	17 (18.3)
Incidence of GFI	24 (25.8)

Data are presented as *n* (%) or median (interquartile range)

*BMI* Body mass index, *RAC* Revised atlanta criteria, *APACHE II* Acute physiology and chronic health evaluation II, *AKI* Acute kidney injury, *GFI* Gastric feeding intolerance

**Table 2:** Baseline characteristics and clinical outcomes in patients with and without GFI

	GFI group (*n* = 24)	Non-GFI group (*n* = 69)	*P*
Age	40 (33–48)	40 (33–48)	0.954
Male	13 (54.2)	50 (72.5)	0.099
BMI	26.2 (23.6–28.4)	26.1 (24.1–30.1)	0.706
APACHE II	10 (7–11)	7 (5–10)	0.003
Time from onset of abdominal pain to study hospital admission	4 (1–4)	3 (2–5)	0.771
RAC			< 0.001
Moderate	8 (33.3)	60 (87)	
Severe	16 (66.7)	9 (13)	
Etiology			0.565
Hypertriglyceridemia	12 (50)	31 (44.9)	
Biliary	10 (41.7)	26 (37.7)	
Others	2 (8.3)	12 (17.4)	
Systemic complications at hospital admission			
Respiratory failure	16 (66.7)	23 (33.3)	0.004
AKI	5 (20.8)	2 (2.9)	0.016
Shock	2 (8.3)	1 (1.4)	0.162
Clinical outcomes			
Energy target-reaching rate between day3-day7	12 (50)	61 (88.4)	< 0.001
Hospital mortality	1 (4.2)	0 (0)	0.258
Length of hospital stay, day	18 (9–31)	6 (4–9)	< 0.001
Pancreaticocutaneous fistula	1 (4.2)	0 (0)	0.258
Abdominal bleeding	2 (8.3)	0 (0)	0.065
IPN	3 (12.5)	0 (0)	0.016
New receipt of organ support therapy			
MV	2 (8.3)	1 (1.4)	0.162
Vasopressors	1 (4.2)	0 (0)	0.258
Mean total inpatient hospital costs, k¥	88.2 (42.2–16.2)	29.4 (22.3–42.0)	< 0.001

Data are presented as *n* (%) or median (interquartile range)

*BMI* Body mass index, *RAC* Revised atlanta criteria, *APACHE II* Acute physiology and chronic health evaluation II, *AKI* Acute kidney injury, *GFI* Gastric feeding intolerance, IPN Infected pancreatic necrosis, MV Mechanical ventilation

### Risk factors of gastric feeding intolerance

In the univariate analysis, the presence of respiratory failure and APACHE II ≥ 8 at admission were associated with GFI (Additional file 1: Table S1). The multicollinearity analysis showed that the VIFs of respiratory failure and APACHE II score were 1.107 and 1.196, respectively. In multivariable analysis, respiratory failure (odds ratio = 3.135, 95% CI: 1.111–8.848, *P* = 0.031) was associated with GFI, whereas the APACHE II did not (Table 3). In the sensitivity analysis, the imbalance in the characteristics (age, gender, etiology and time from the onset of abdominal pain to hospital admission) between the GFI and non-GFI groups was significantly minimised with SMD < 0.1 (Additional file 1: Fig. S2) after PSM. Nevertheless, patients in the GFI group had a higher proportion of respiratory failure, lower energy target-reaching rate between day3-day7, longer length of hospital stay, and higher cost when compared with the non-GFI group (Additional file 1: Table S2).

**Table 3:** Multivariable logistic regression analysis for GFI

Variables	OR (95% CI)	*P* value
Age	0.981 (0.942–1.022)	0.369
Respiratory failure	3.135 (1.111–8.848)	0.031
APACHE II		
< 8	1 (reference)	–
≥ 8	3.423 (1.133–10.343)	0.05

GFI Gastric feeding intolerance, *APACHE* II Acute physiology and chronic health evaluation II

### Nutrition therapy

**Fig. 2:**
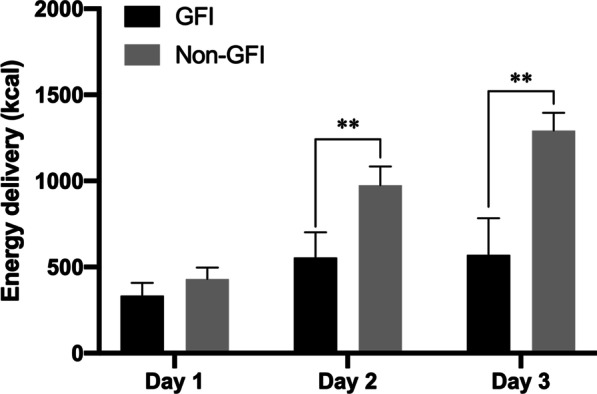
Energy delivery within the first three days after nasogastric tube placement in GFI group and non-GFI group. All patients were categorised into GFI group (dark boxes) and non-GFI group (light boxes) groups (** *p* < 0.001). Error bars indicate 95% confidence intervals

The median time of switching to NJ feeding in the GFI group was five days (interquartile range: 4–7 days) after admission (Table 4). Abdominal pain was the most common gastrointestinal symptom in patients intolerant to gastric feeding, with an incidence of 91.7%. The mean daily energy delivery in the following three days after switching to NJ feeding was significantly increased than the first three days after initiation of NG feeding in patients with GFI [920.83 (493.33–1326) vs. 465 (252.25–556.67) kcal, *P* < 0.001].The energy delivery within the first three days after nasogastric tube placement is presented in Fig. 2. Patients without GFI had a significantly higher calorie intake on day 2 and day 3 than those with GFI.

**Table 4:** Nutrition therapy variables in patients with GFI

Variables	
Mean daily energy delivery, kcal	
The first three days after initiation of nasogastric feeding	465 (252.25–556.67)
The following three days after switching to nasojejunal feeding	920.83 (493.33–1326)
Intolerance symptom to gastric feeding	
Abdominal pain	22 (91.7)
Vomiting	6 (25)
IAP > 15 mmHg	3 (12.5)
GRV ≥ 250 ml/6 h	1 (4)
Time to switch to NJT after hospitalization, days	5 (4–7)

Data are presented as *n* (%) or median (interquartile range)

*GFI* Gastric feeding intolerance, *IAP* Intra-abdominal pressure, *GRV* Gastric residual volume

## Discussion

To the best of our knowledge, this is the first study to evaluate the incidence and risk factors of nasogastric feeding intolerance in moderately-severe to severe AP. Our study found that GFI is common in moderately-severe to severe AP patients with an incidence of 25.8%, and the presence of respiratory failure may increase the risk of GFI. Furthermore, the time to discharge alive from the hospital was longer in the GFI group, though the patients with GFI were sicker at admission, evidenced by a higher APACHEII score.

The optimal route of early EN in AP patients remains unclear. Three randomised controlled trials (RCTs) involving 157 patients have demonstrated similar outcomes regarding tolerance, complications rates, and mortality between NG or NJ feeding [1–3]. Several reasons could weaken the reliability of the conclusion. First, all three trials had a limited sample size, and the eligibility criteria differ among the three RCTs. Second, the feeding protocol and target applied in the three trials vary. Moreover, the position of the NJ tube tip was not precisely reported in these studies [22]. In our study, all patients in the GFI group (switching to the NJ feeding) had NJ tubes placed into the mid-distal jejunum confirmed by the abdominal radiograph.

Although NG feeding is recommended as the first‑line choice for EN in AP patients because of the advantages of lower cost and easy insertion, patients complicated by organ failure and/or pancreatic necrosis/fluid collections were at risk of impaired gastric emptying or gastric outlet obstruction, making them vulnerable to GFI [23, 24]. However, data on GFI incidence in AP patients are scarce, especially in more severe types of disease. Recently, a retrospective study involving exclusively moderately-severe AP patients found that GFI (defined as when at least 20 kcal/kg BW/day via enteral route cannot be reached within 72  h of feeding attempt) occurred in 34% of patients receiving NG feeding [25], which is higher than our results. Even though the different study populations and definitions of GFI limit the comparability, both studies suggest that the incidence of GFI is considerable in patients with more severe AP.

The presence of GFI could decrease the amount of EN delivery and prevent feeding goals from being achieved, as shown in our study. Moreover, energy inadequacy might be associated with poor clinical outcomes, including prolonged mechanical ventilation and increased risk of infection [26–28]. Futhermore, patients receiving NG feeding are more likely to have the risk of reflux and aspiration when complicated with GFI, which may increase the aspiration-related complications [11, 29–32].

The development of organ failure is one of the major determinants of mortality in patients with AP [33]. Previous studies have shown that respiratory failure is the most common type of organ failure in AP and is associated with high mortality [33, 34]. In our study, respiratory failure was significantly associated with GFI. Similar findings were reported in critically ill patients with acute respiratory failure, and the leading gastrointestinal symptoms included vomiting and diarrhoea [35]. Analgesic opioid drugs used during MV were considered the potential causes [36, 37]. Thus, AP patients with early respiratory failure might benefit from the early NJ feeding because of better early EN implementation.

This study had several limitations. Due to the retrospective unicentric design and the limited sample size, a causal relationship cannot be inferred, and the results should be interpreted with caution. Moreover, centre experience and volume might impact severity/complications of the disease [38]. Since our centre is one of the largest referral sites for AP in China, the majority of the screened patients (346/561, 61.7%) were admitted later than seven days from the onset of abdominal pain in the present study, and more than half of the excluded patients had SAP (SAP (265/468, 56.6%), MSAP (117/468, 25%), MAP (86/468, 28.4%), respectively). In addition, the comparisons for clinical outcomes were just descriptive without appropriate adjustment, and the incidence of aspiration was not reported in our study due to unreliable recordings. Last, the diagnosis of GFI and the decision to switch was made by the treating physician, which inevitably brought some bias into our results due to the subjectivity.

The strength of this study is that, although explorative, this is the first study to investigate the risk factors for GFI in moderately-severe to severe AP, and the results may help identify a specific cohort of patients who may benefit from NJ feeding as the first line for EN delivery. A randomised controlled trial is therefore warranted to evaluate the clinical impact of primary NJ feeding in patients with AP complicated by early respiratory failure.

## Conclusion

Our study showed that GFI is common in moderately-severe to severe AP patients, with an incidence of 25.8%. The presence of respiratory failure may increase the risk of GFI. Further randomised clinical trials are needed to assess the impact of jejunal feeding as the initial EN delivery route in AP patients with early respiratory failure.

## Supplementary Information

**Additional file 1: Table S1**. Univariate logistic regression analysis for GFI. **Table S2.** Baseline characteristics and clinical outcomes in patients with and without GFI after propensity score matching. **Fig. S1** Time to discharge alive from the hospital within 30 days after hospital admission. **Fig. S2** Standardised mean difference of variables before and after propensity score matching

## Data Availability

The datasets are not publicly available due to privacy or ethical restrictions but are available from the corresponding author on reasonable request.

## Abbreviations

EN: Enteral nutrition
AP: Acute pancreatitis
NG: Nasogastric
NJ: Nasojejunal
GFI: Gastric feeding intolerance
TPN: Total parenteral nutrition
SAP: Severe acute pancreatitis
MAP: Mild acute pancreatitis
MSAP: Moderate-severe acute pancreatitis
CSAP: Center of severe acute pancreatitis
RAC: Revised atlanta criteria
BMI: Body mass index
APACHE II: Acute physiology and chronic health evaluation II
AKI: Acute kidney injury
RCTs: Randomised controlled trials
