# Supplementary material for: Incidence and risk factors of nasogastric feeding intolerance in moderately-severe to severe acute pancreatitis
Source: BMC Gastroenterol. 2022 Jul 2;22:327. doi: 10.1186/s12876-022-02403-w (PMC9250174; doi:10.1186/s12876-022-02403-w)
Supplement: Supplementary file 1 — Additional file 1: Table S1. Univariate logistic regression analysis for GFI. Table S2. Baseline characteristics and clinical outcomes in patients with and without GFI after propensity score matching. Fig. S1 Time to discharge alive from the hospital within 30 days after hospital admission. Fig. S2 Standardised mean difference of variables before and after propensity score matching [file 12876_2022_2403_MOESM1_ESM.docx]

**Additional information to**

**Incidence and risk factors** **of nasogastric feeding intolerance in moderately-severe to severe acute pancreatitis**

Jiajia Lin, MD ^1, #^, Cheng Lv, MD ^1, #^, Cuili Wu, MD ^2, #^, He Zhang, MD ^3^, Zirui Liu, MD ^1^, Lu Ke, PhD ^1, 5^, Gang Li, MD ^1, *^, Zhihui Tong, PhD ^1^, Jianfeng Tu, PhD ^4, *^, Weiqin Li, PhD ^1, 5^

1. Center of Severe Acute Pancreatitis (CSAP), Department of Critical Care Medicine, Jinling Hospital, Medical School of Nanjing University, Nanjing, 210002, PR China.

2. Department of General Surgery, Jinling Hospital, Medical School of Nanjing University, Nanjing, 210002, PR China.

3. Department of Critical Care Medicine, Jinling Hospital, Medical School of Southeast University, Nanjing, 210002, PR China.

4.Department of Emergency Medicine, Zhejiang Provincial People's Hospital, Hangzhou medical college, Hangzhou, Zhejiang 310014, PR China.

5. National Institute of Healthcare Data Science, Nanjing University, Nanjing, China.

**CONTENTS**

Table S1. Univariate logistic regression analysis for GFI.……….……….…………….…………….……….…….…………….…………Page 2

Table S2. Baseline characteristics and clinical outcomes in patients with and without GFI after propensity score matching.……….………Page 3

Figure S1. Time to discharge alive from the hospital within 30 days after hospital admission ..…………….……….……….…….……....Page 5

Figure S2. Standardized mean difference (SMD) of variables before and after propensity score matching ..…………….…………..……....Page 6

Table S1. Univariate logistic regression analysis for GFI.

| Variables | OR (95% CI) | P value |
| --- | --- | --- |
| Age | 0.997 (0.961-1.034) | 0.878 |
| Male | 0.449 (0.172-1.174) | 0.103 |
| BMI | 0.949 (0.856-1.053) | 0.325 |
| Respiratory failure | 4 (1.493-10.714) | 0.006 |
| **Etiology** |  |  |
| Non-biliary | 1 (reference) |  |
| Biliary | 1.181 (0.459-3.043) | 0.73 |
| **APACHE II** |  |  |
| <8 | 1 (reference) | - |
| ≥8 | 3.556 (1.305-9.694) | 0.013 |

Table S2. Baseline characteristics and clinical outcomes in patients with and without GFI after propensity score matching

|  | GFI group  (n=24) | Non-GFI group  (n=24) | P |
| --- | --- | --- | --- |
| Age | 40 (33-48) | 43 (32-48) | 0.812 |
| Male | 13 | 13 |  |
| BMI | 26.24 (23.58-28.40) | 26.21 (23.81-29.75) | 0.805 |
| APACHE II | 10 (7-11) | 8 (5-10) | 0.078 |
| Time from onset of abdominal pain to hospital admission | 4 (1-4) | 3 (2-4) | 0.858 |
| **RAC** |  |  | <0.001 |
| Moderate | 8 (33.3) | 21 (87.5) |  |
| Severe | 16 (66.7) | 3 (12.5) |  |
| **Etiology** |  |  | 1 |
| Hypertriglyceridemia | 12 (50) | 13 (54.2) |  |
| Biliary | 10 (41.7) | 9 (37.5) |  |
| Others | 2 (8.3) | 2 (8.3) |  |
| **Systemic complications at hospital admission** |  |  |  |
| Respiratory failure | 16 (66.7) | 7 (29.2) | 0.02 |
| AKI | 5 (20.8) | 0 (0) | 0.05 |
| Shock | 2 (8.3) | 0 (0) | 0.489 |
| **Clinical outcomes** |  |  |  |
| Energy target-reaching rate between day3-day7 | 12 (50) | 20 (83.3) | 0.03 |
| Hospital mortality | 1 (4.2) | 0 (0) | 1 |
| Length of hospital stay, day | 18 (9-31) | 7 (5-9) | <0.001 |
| Pancreaticocutaneous fistula | 1 (4.2) | 0 (0) | 1 |
| Abdominal bleeding | 2 (8.3) | 0 (0) | 0.489 |
| IPN | 3 (12.5) | 0 (0) | 0.234 |
| **New receipt of organ support therapy** |  |  |  |
| MV | 2 (8.3) | 1 (4.2) | 1 |
| Vasopressors | 1 (4.2) | 0 (0) | 1 |
| Mean total inpatient hospital costs, k¥ | 88.19 (42.26-161.98) | 29.71 (23.34-39.75) | <0.001 |

Data are presented as n (%) or median (interquartile range).

BMI, Body Mass Index; RAC, Revised Atlanta Criteria; APACHE II, Acute Physiology and Chronic Health Evaluation II; AKI, Acute Kidney Injury; GFI, Gastric Feeding Intolerance; IPN, Infected Pancreatic Necrosis; MV, Mechanical Ventilation.

Figure S1. Time to discharge alive from the hospital within 30 days after hospital admission.


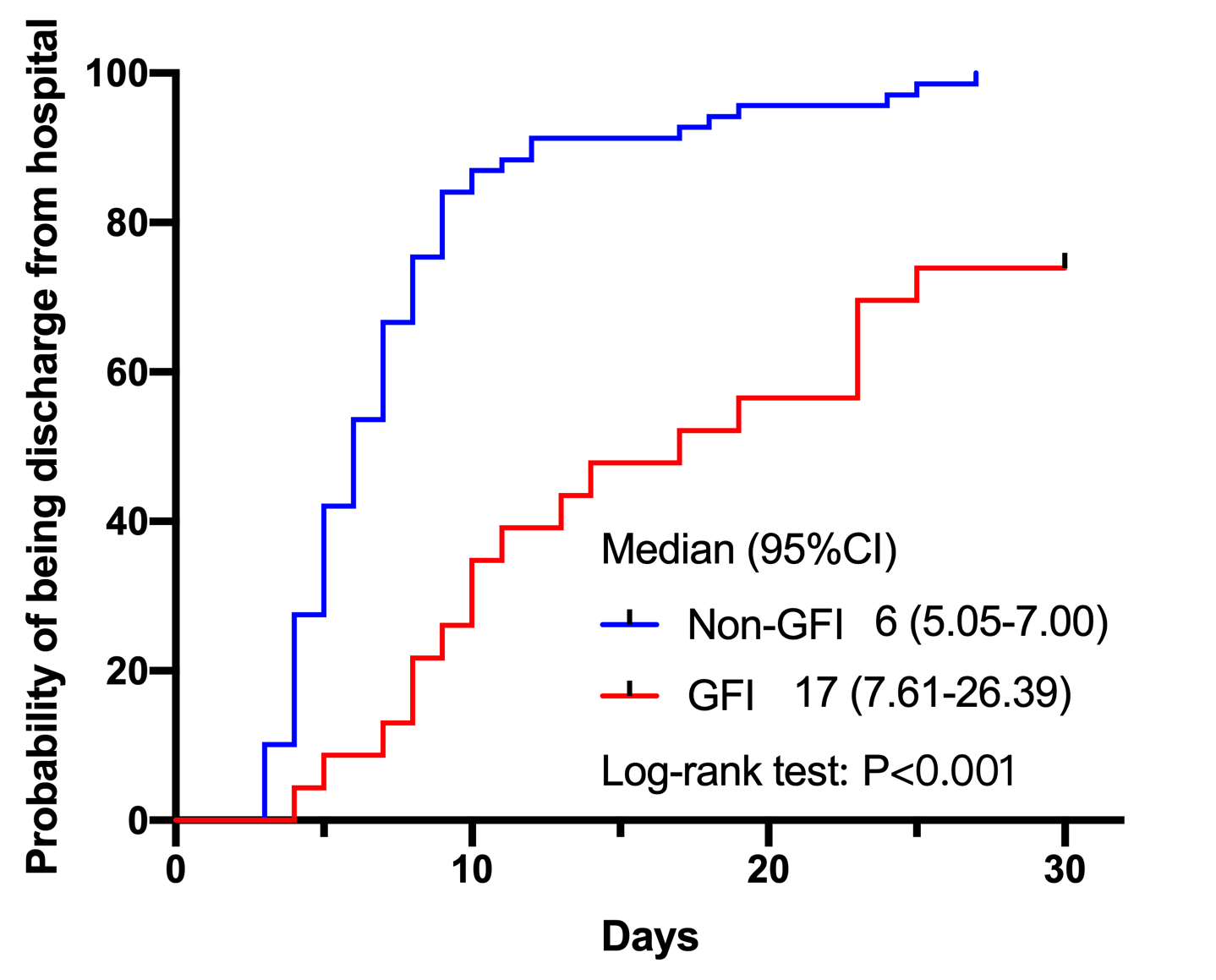


Kaplan-Meier estimates of probability of patients being discharge from hospital within 30 days. Higher values represent a higher probability of being discharge from hospital at a certain time point; in hospital deaths and transfers were censored at time of those events.

Figure S2: Standardised mean difference (SMD) of variables before and after propensity score matching


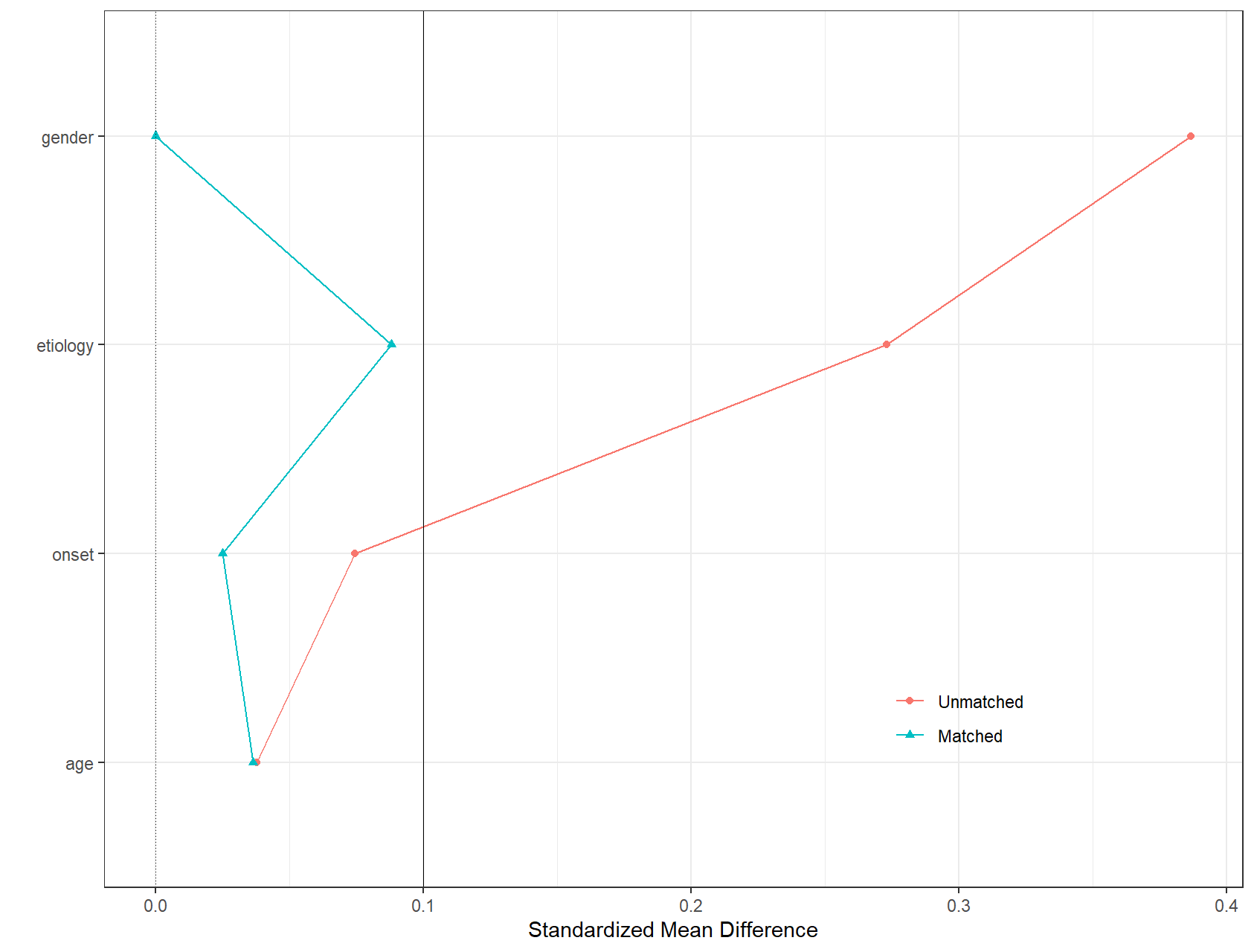


onset denotes the time from onset of AP to hospital admission.
